# Assessment of attitudes and practices towards COVID-19 pandemic: a survey on a cohort of educated Syrian population

**DOI:** 10.1186/s42506-023-00142-8

**Published:** 2023-09-04

**Authors:** Lina Albitar, Ghalia Aboualchamat

**Affiliations:** 1 Department of Pharmaceutics, Faculty of Pharmacy, Arab International University, Damascus, Syria; 2 Department of Biology, Faculty of Science, Damascus University, Damascus, Syria; 3 Centre for Epidemiological and Biological Studies, Damascus University, Damascus, Syria

**Keywords:** Attitude, COVID19, Coronavirus disease, Practice, Vaccine, Syria

## Abstract

**Background:**

Coronavirus disease (COVID-19) caused the death of millions of people and affected the lives of hundreds of millions worldwide. The WHO recommendations aimed mainly to reduce transmission, minimize infection, and get people vaccinated. Nevertheless, opinions and attitudes about the disease varied. In this study, we evaluated personal attitudes and practices of a cohort of an educated Syrian population, after several waves of infection with COVID-19 and the release of different types of vaccines.

**Methods:**

A cross-sectional internet-based survey was launched in January 2022.The survey queried the participants’ personal experience, attitudes, practices towards COVID-19, and vaccination.

**Results:**

The study included 408 individuals. The respondents were mainly females (72.6%), 20–29 years old (39.2%), and college graduates (59.3%). A large proportion (89.7%) reported having been infected at least once during the pandemic; a significant association was found with age (*p* = *0.001*). Nearly half of the respondents got vaccinated; the majority were > 40 years old. Opinions differed regarding the effectiveness and safety of the vaccines; only a small percentage of the participants (17.4%) thought all vaccines were effective and safe. Remarkably, the level of education did not significantly dominate the participants’ attitudes or practices towards the COVID-19 pandemic. Approximately half of the respondents (44.9%) stated their lives were affected by the pandemic and over the third were worried (38%). A significant association was detected with gender in favour of females. Most of the participants have taken at least one precautionary measure to limit the infection.

**Conclusion:**

The level of education did not significantly dominate the participants’ attitudes or practices towards the COVID-19 pandemic. Female respondents were more cautious, concerned and committed to taking precautionary measures regardless of their education level. However, their unwillingness to receive the vaccine raises significant concerns. Efforts should be made to emphasize the importance of immunization, the safety and effectiveness of vaccines, and encourage vaccination among individuals.

**Supplementary Information:**

The online version contains supplementary material available at 10.1186/s42506-023-00142-8.

## Introduction

The first outbreak of the Severe Acute Respiratory Syndrome Coronavirus 2 (SARS-CoV-2) occurred on December 31, 2019 in Wuhan City, China [1]. The virus that causes Coronavirus disease 2019 (COVID-19), has spread rapidly and formed a serious health concern worldwide [2]. According to the WHO, by April 29, 2023, the confirmed cases of COVID-19 reached over 764 million, including more than 6.9 million deaths [1]. Intensive studies have been conducted to track the outbreaks of clinically significant mutant viruses, expected to affect disease severity, named variants of interest (VOI). Additional studies investigated variants that increase transmissibility and virulence or reduce effectiveness in prevention, diagnosis, and treatment, which are called variants of concern (VOC) [3].

Scientists working on COVID-19 have faced significant challenges in finding the appropriate treatment and developing effective vaccines, in an effort to minimize symptom severity, hospitalization and mortality rates [4–7]. Hundreds of vaccines have been rapidly developed, which differed in their efficacy against COVID-19 variants and in their side effects [8]. The Food and Drug Administration (FDA) and/or the WHO approved several vaccines [9]. According to many studies vaccine acceptance differed broadly between countries and between individuals with different sociodemographic backgrounds [10, 11] In addition, different attitudes and practices towards the pandemic have been observed worldwide [12, 13].

In Syria, the first confirmed Coronavirus case was declared in March 2020 [14]. Since then, health authorities have taken various important precautionary measures to limit the spread of the infection. Measures started with educating the public, recommending people to stay at home, and imposing night curfews. Later, the measures expanded to inter-city travel restrictions, closing of schools and universities, and banning public gatherings. Finally, the authorities issued a complete lockdown. Since educated individuals exert influence on their communities, it is highly important to explore their attitudes and practices, especially at the time of pandemic. In this study, we aimed to evaluate the attitudes, practices and the safety measures taken by a cohort of educated Syrian population against the COVID-19 pandemic.

## Methods

### Study design and data collection

An internet-based survey was designed by the authors, using the Google Forms. The survey’s questions were in Arabic and were evaluated by two experts for relevance and clarity. A draft of the survey was piloted on 30 participants to assess the survey’s simplicity, clarity, and ease to understand. The validated survey was uploaded to websites and private groups on social media mainly Facebook and WhatsApp groups. The survey began with a brief introduction and the aim of the study, followed by 22 “close-ended” and “multiple-choice” questions (Supplement 1), as follows:

- Questions Q1-Q8: gathered general information on the participants and explored participants’ personal experiences with the Coronavirus disease.
- Questions Q9-Q18: inspected the participants’ behaviour, attitudes and practices regarding personal protective equipment (PPE) and safety measures, towards the Coronavirus disease.
- Questions Q19-Q 22: explored participants’ opinions and attitudes towards COVID-19 vaccines and getting vaccinated.

The survey was accessible online for 10 weeks (January 3^rd^ -March 16^th^, 2022). Taking the survey was voluntary; nevertheless, the respondent’s consent of participation was taken at the end of the introduction section. Middle school education level or higher was considered as the inclusion criteria.

### Sample size

The sample size was estimated using the equation: *n* = z^2^. [p *q]/d2 [15], z is the Z-score; 1.96 for 5% significance levels, P is the estimated proportion of the studied construct, q = 1-P, and d is the margin of error (5%). The minimum required sample was 384.

### Statistical analysis

The analyses were accomplished using SPSS, Version 25. Descriptive and comparative data were presented as percentages. Nonparametric Kruskal–Wallis, Mann–Whitney and Chi-square tests were used where applicable. For all tests *p value* < 0.05 was considered statistically significant.

## Results

### General description of the participants

A total of 408 respondents took part in the study. The majority of the respondents were females (72.6%), 20–29 years old (39.2%) and college graduates (59.3%). The confirmed cases of COVID-19 infection were 192, mostly females (140). Data revealed significant correlation between infection and age groups (*p* = 0.001), while no significant association with gender nor with education level were found. Furthermore, 20.1% of the participants confirmed being re-infected by COVID-19 and 27.2% suspected being infected more than once. However, the majority of the participants (68.7%) did not confirm their COVID-19 infection by PCR test. Of note, the participants aged > 40 years old sought medical confirmation of the infection more than the other age groups. Nearly half of our respondents (49.3%) got vaccinated against COVID-19, mostly those over 40 years old. More than 20% of the respondents either opposed vaccination or acknowledged not being ready yet to get vaccinated and 8.1% were neutral to the idea. Significant associations were found with age and with education levels (*p* values < 0.001) (Table 1).

**Table 1:** The reported occurrence of Corona virus infection, re-infection, and the vaccination status among the sample of educated Syrian participants in relation to their socio-demographic characteristics, 2021

		**Gender**		**Age**				**Education**			
		**Male (%)**	**Female (%)**	** < 20 (%)**	**20–29 (%)**	**30–40 (%)**	** > 40 (%)**	**Middle School (%)**	**High School (%)**	**Graduates (%)**	**Postgraduate (%)**
**Previous COVID-19 infection**											
Yes	192 (47%)	52 (46.4)	140 (47.3)	15 (34.1)	63 (39.4)	32 (43.8)	82 (62.6)	4 (40.0)	16 (47.1)	109 (45.0)	63 (51.6)
Maybe	157 (38.5%)	41 (36.6)	116 (39.2)	16 (36.4)	76 (47.5)	30 (41.1)	35 (26.7)	5 (50.0)	12 (35.3)	95 (39.3)	45 (36.9)
No	59 (14.5%)	19 (17.0)	40 (13.5)	13 (29.5)	21 (13.1)	11 (15.1)	14 (10.7)	1 (10.0)	6 (17.6)	38 (15.7)	14 (11.5)
**Total**	**408**	112	296	44	160	73	131	10	34	242	122
*Pearson Chi-Square* *P - value*		0.663		< 0.001*				0.834			
**Recurrence of COVID-19 infection**											
Yes	77 (20.1%)	17 (17.0)	60 (21.2)	9 (23.7)	31 (20.3)	14 (20.3)	23 (18.7)	2 (22.2)	7 (21.9)	45 (20.0)	23 (19.7)
Maybe	104 (27.2%)	29 (29.0)	75 (26.5)	7 (18.4)	46 (30.1)	18 (26.1)	33 (26.8)	4 (44.4)	3 (9.4)	53 (23.6)	44 (37.6)
No	202 (52.7%)	54 (54.0)	148 (52.3)	22 (57.9)	76 (49.7)	37 (53.6)	67 (54.5)	3 (33.3)	22 (68.8)	127 (56.4)	50 (42.7)
**Total**	**383**^**a**^	100	283	38	153	69	123	9	32	225	117
*Pearson Chi-Square* *P - value*		0.651		0.870				0.016*			
**Medical confirmation of COVID-19 infection**											
Yes	120 (31.3%)	38 (38.0)	82 (29.0)	6 (15.8)	27 (17.6)	27 (39.1)	60 (48.8)	3 (30.0)	10 (31.3)	62 (27.6)	45 (38.8)
No	263 (68.7%)	62 (62.0)	201 (71.0)	32 (84.2)	126 (82.4)	42 (60.9)	63 (51.2)	7 (70.0)	22 (68.8)	163 (72.4)	71 (61.2)
**Total**	**383**^**a**^	100	283	38	153	69	123	10	32	225	116
*Pearson Chi-Square* *P - value*		0.094		< 0.001*				0.212			
**Vaccination against COVID-19**											
Yes	201 (49.3)	63 (56.3)	138 (46.6)	7 (15.9)	62 (38.8)	47 (64.4)	85 (64.9)	0 (0.0)	6 (17.6)	114 (47.1)	81 (66.4)
Not ready yet	92 (22.5)	18 (16.1)	74 (25.0)	14 (31.8)	53 (33.1)	9 (12.3)	16 (12.2)	4 (40.0)	12 (35.3)	59 (24.4)	17 (13.9)
Never	82 (20.1)	21 (18.8)	61 (20.6)	15 (34.1)	34 (21.3)	12 (16.4)	21 (16.0)	5 (50.0)	9 (26.5)	50 (20.7)	18 (14.8)
Neutral	33 (8.1)	10 (8.9)	23 (7.8)	8 (18.2)	11 (6.9)	5 (6.8)	9 (6.9)	1 (10.0)	7 (20.6)	19 (7.9)	6 (4.9)
**Total**	**408**	112	296	44	160	73	131	10	34	242	122
*Pearson Chi-Square* *P - value*		0.197		< 0.001*				0.001*			

^*^Significant at the 0.05 level

^a^The total does not add to 408 because some questions were not applicable

The majority of the participants (67.9%) reported that at least one person in their household had caught COVID-19. A significant association was revealed between the infection of the respondents and infection of any of their household members (*p* < 0.001). Additionally, a significant association was detected between the reinfection of the respondents and infection of their household members (*p* = 0.012) (Table 2).

**Table 2:** Association between the participants’ infection with COVID-19 and the infection of household members

	**Infection of household members with COVID-19**		Pearson Chi-Square *P-value*
	Yes /maybe (%)	No (%)	
**Participants infection with COIVD-19**			
Yes /maybe	315 (89.7)	34 (59.6)	< 0.001*
No	36 (10.3)	23 (40.4)	
**Recurrence of the infection in participants**			
Yes /maybe	168 (49.6)	13 (29.5)	0.012*
No	171 (50.4)	31 (70.5)	

^*^Significant at the 0.05 level

**Fig. 1:**
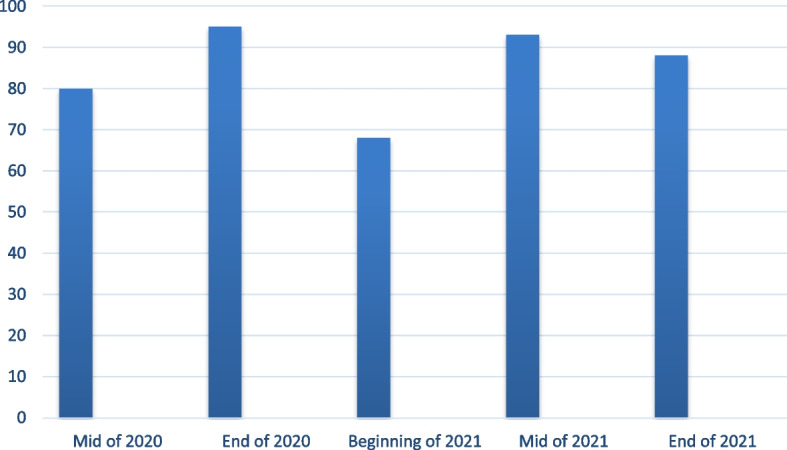
Frequency of COVID-19 infection among the educated Syrian participants (2020–2021). The highest COVID-19 infections occurred in late 2020 (95 participants) and mid-2021 (93 participants)

**Fig. 2:**
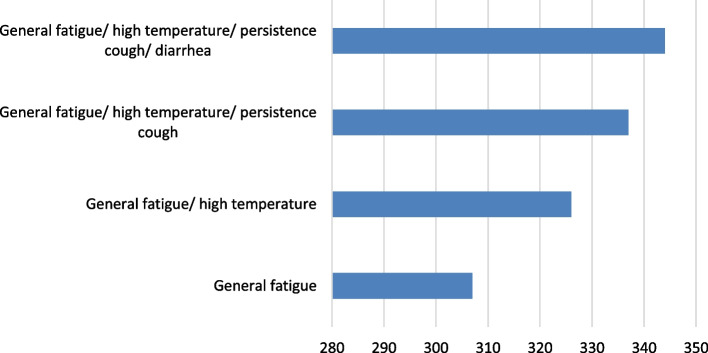
The common symptoms experienced by the educated Syrian respondents. General fatigue was the most prevalent symptom

The highest numbers of COVID-19 infections occurred in late 2020 and mid 2021 (Fig. 1). Overall, general fatigue was the most common symptom experienced by more than three quarters of the respondents (Fig. 2).

### Personal attitudes

Over half of the participants (56.1%) responded positively towards self-isolation. A significant association was found between genders, age and education in favour of females, > 40 years old, and college graduates respectively. Furthermore, for approximately half of the respondents (44.9%), the pandemic has affected their personal lives and relationships with others. A significant association was found with gender in favor of females.

When the participants were asked about their attitude towards wearing face masks, more than half of the respondents confirmed its necessity (51.7%), while over one quarter of them (26.5%) declared not being committed to wearing face masks, despite confirming its importance. A significant association was found with gender in favour of females and with age, in favour of the participants > 40 years old.

Furthermore, approximately 75% of the participants stated dealing cautiously with an infected household member. In contrast, a small percentage of them choose either to deal indifferently or avoid dealing at all with infected patients (16.1% and 9.7%, respectively). A significant difference was detected with age (Table 3).

**Table 3:** Attitude of educated Syrian adults towards COVID-19 pandemic, 2022

**Attitude**		**Gender**		**Age**				**Education**			
		**Male (%)**	**Female (%)**	**< 20 (%)**	**20–29 (%)**	**30–40 (%)**	**> 40 (%)**	**Middle School (%)**	**High School (%)**	**Graduates (%)**	**Postgraduate (%)**
**Self-isolation when feeling sick**											
Yes	229 (56.1)	56 (50)	173 (58.4)	18 (40.9)	94 (58.8)	34 (46.6)	83 (63.4)	6 (60.0)	18 (52.9)	141 (58.3)	64 (52.5)
Maybe	19 (4.7)	9 (8)	10 (3.4)	5 (11.4)	6 (3.8)	5 (6.8)	3 (2.3)	0 (0.0)	5 (14.7)	11 (4.5)	3 (2.5)
No	37 (9.1)	17 (15.2)	20 (6.8)	3 (6.8)	10 (6.3)	10(13.7)	14 (10.7)	1 (10.0)	1 (2.9)	14 (5.8)	21 (17.2)
As need	123 (30.1)	30 (26.8)	93 (31.4)	18 (40.9)	50 (31.3)	24 (32.9)	31 (23.7)	3 (30.0)	10 (29.4)	76 (31.4)	34 (27.9)
**Total**	**408**										
*Pearson Chi-Square* *P - value*		0.008*		0.027*				0.006*			
**Pandemic impact on life**											
Yes	183 (44.9)	47 (42.0)	136 (45.9)	15 (34.1)	72 (45.0)	30 (41.1)	66 (50.4)	5 (50.0)	10 (29.4)	110 (45.5)	58 (47.5)
No	120 (29.4)	44 (39.3)	76 (25.7)	11 (25.0)	47 (29.4)	25 (34.2)	37 (28.2)	4 (40.0)	8 (23.5)	68 (28.1)	40 (32.8)
Maybe	61 (15.0)	12 (10.7)	49 (16.6)	14 (31.8)	26 (16.3)	9 (12.3)	12 (9.2)	1 (10.0)	11 (32.4)	39 (16.1)	10 (8.2)
Neutral	44 (10.8)	9 (8.0)	35 (11.8)	4 (9.1)	15 (9.4)	9 (12.3)	16 (12.2)	0 (0.0)	5 (14.7)	25 (10.3)	14 (11.5)
**Total**	**408**										
*Pearson Chi-Square* *P - value*		0.039*		0.07				0.064			
**Attitude towards face masks and its effectiveness**											
Must be worn (%)	211 (51.7)	60 (53.6)	151(51.0)	20 (45.5)	72 (45.0)	40 (54.8)	79 (60.3)	6 (60.0)	18 (52.9)	115 (47.)	72 (59.0)
Does not protect (%)	89 (21.8)	31 (27.7)	58 (19.6)	12 (27.3)	31 (19.4)	19 (26.0)	27 (20.6)	3 (30.0)	10 (29.4)	51 (21.1)	25 (20.5)
Confirm importance not committed (%)	108 (26.5)	21 (18.8)	87 (29.4)	12 (27.3)	57 (35.6)	14 (19.2)	79 (19.1)	1 (10.0)	6 (17.6)	76 (31.4)	25 (20.5)
**Total**	**408**										
*Pearson Chi-Square* *P - value*		0.049*		0.023*				0.148			
**Dealing with an infected household member**											
Cautiously (%)	276 (74.2)	69 (69.7)	207 (75.8)	30 (76.9)	110 (74.3)	41 (61.2)	95 (80.5)	8 (80.0)	22 (71.0)	161 (72.9)	85 (77.3)
Indifferently (%)	60 (16.1)	23 (23.2)	37 (13.6)	7 (17.9)	16 (10.8)	21 (31.3)	95 (13.6)	2 (20.0)	5 (16.1)	33 (14.9)	20 (18.2)
Avoided (%)	36 (9.7)	7 (7.1)	29 (10.6)	2 (5.1)	22 (14.9)	5 (7.5)	7 (5.9)	0 (0.0)	4 (12.9)	27 (12.2)	5 (4.5)
**Total**	**372**										
*Pearson Chi-Square* *P - value*		0.063		0.001*				0.357			

^*^Significant at *p*< 0.05

The anxiety level of the participants was measured on a five-point scale as follows: 1 = very worried, 2 = worried, 3 = neutral, 4 = unworried and 5 = not worried at all. Our data showed that 4.2% of the respondents were very worried, 38% were worried 11.3% were neutral, 27.9% were unworried, and 18.6% were unworried at all. Significant differences were found between genders (*p* = *0.021*) and between age groups (*p* = *0.01*). Male participants were less concerned compared to females; the average anxiety scores were 3.42 ± 1.3 and 3.10 ± 1.2, respectively. Moreover, the participants belonging to the age group 30–40 years old were unworried (3.59 ± 1.3); whereas the participants from the other age groups were neutral (Table 4).

**Table 4:** Differences in the anxiety level towards COVID-19 pandemic amongst the educated Syrian participants, 2021

**Variables**	**Anxiety level**					
	**N**	**Mean ± SD**	**Mean Rank**	***P*-value**		***P-*value**
**Gender**						
Male	112	3.42 ± 1.3	225.46	0.021^a^*		
Female	296	3.10 ± 1.2	196.57			
**Age (in years)**						
< 20	44	3.27 ± 1.2	212.44	0.010^b^*	1–2	0.526
20–29	160	3.14 ± 1.2	200.11		1–3	0.143
					1–4	0.158
30–40	73	3.59 ± 1.3	241.05		2–3	0.009*
					2–4	0.303
> 40	131	2.99 ± 1.2	186.82		3–4	0.001*
**Education level**						
Middle school	10	3.10 ± 1.4	199.20	0.572^b^		
High School	34	3.29 ± 1.2	213.63			
Graduates	242	3.12 ± 1.2	198.25			
Postgraduate	122	3.30 ± 1.2	214.80			

1- < 20 years old; 2- 20–29 years old; 3- 30–40 years old; 4- > 40 years old

^*^Significant at the 0.05 level

^a^Mann-Whitney Test, ^b^Kruskal-Wallis Test

### Personal practices

Less than half of the participants (46.5%) reported coughing or sneezing in their face masks, while a small number never did this practice (23.7%). The majority of the respondents (62.1%) acknowledged replacing or washing their face masks regularly. No significant differences were detected between participants with regard to gender, age, or education levels. In addition, almost half of the respondents (49.5%) stated that they were accustomed to pursuing good personal hygiene habits before the COVID-19 pandemic, whereas a small number declared that they never did this practice (7.6%). A significant difference was found with gender (Table 5). Our statistical analysis showed significant differences between genders in avoiding close contact with patients or the elderly, in keeping social distances between age groups, and in cleaning high-touched surfaces between genders (Table 5).

**Table 5:** Precautionary practices done by the educated Syrian participants according to gender, age and education

	Cough or sneeze in face masks			Replace or wash face mask regularly					Pursuing personal hygiene habits before the pandemic				Avoid close contact with elderly or patients		Keep a distance from others		Cleaning high-touch surfaces			
	Yes (%)	No (%)	Sometimes (%)	Always (%)		No (%)	Sometimes (%)	When dirty (%)	Always (%)	Often (%)	Sometimes (%)	No (%)	Yes (%)	No (%)	Yes (%)	No (%)	Yes (%)			No (%)
**Male**	51 (48.1)	28 (26.4)	27 (25.5)	61 (58.1)		13 (12.4)	17 (16.2)	14 (13.3)	45 (40.2)	40 (35.7)	19 (17.0)	8 (7.1)	43 (38.4)	69 (61.6)	41 (36.6)	71 (63.4)	49 (43.8)			63 (56.3)
**Female**	130 (45.9)	64 (22.6)	89 (31.4)	181 (63.5)		14 (4.9)	48 (16.8)	42 (14.7)	157 (53.0)	69 (23.3)	47 (15.9)	23 (7.8)	155 (52.4)	141 (47)	98 (33.1)	198 (66.)	177 (59.8)			119 (40.2)
**Total**	181 (46.5)	92 (23.7)	116 (29.8)	242 (62.1)		27 (6.9)	65 (16.7)	56 (14.4)	202 (49.5)	109 (26.7)	66 (16.2)	31 (7.6)	198	210	139	269	226			182
***Pearson Chi-Square*** ***P-value***	0.47			0.08					0.05*				0.01*		0.50		0.004*			
**< 20**	18 (41.9)	10 (23.3)	15 (34.9)	24 (55.8)		2 (4.7)	7 (16.3)	10 (23.3)	24 (54.5)	12 (27.3)	4 (9.1)	4 (9.1)	19 (43.2)	25 (56.8)	11 (25.0)	33 (75.0)	28 (63.6)		16 (36.4)	
**20–29**	67 (44.1)	42 (27.6)	43 (28.3)	99 (64.3)		15 (9.7)	22 (14.3)	18 (11.7)	84 (52.5)	40 (25.0)	25 (15.6)	11 (15.6)	79 (49.4)	81 (50.6)	43 (26.9)	117(73.)	94 (58.8)		66 (41.3)	
**30–40**	44 (62.9)	10 (14.3)	16 (22.9)	47 (68.1)		4 (5.8)	11 (15.9)	7 (10.1)	32 (43.8)	14 (19.2)	21 (28.8)	21 (28.8)	33 (45.2)	40 (54.8)	29 (39.7)	44 (60.3)	36 (49.3)		37 (50.7)	
**> 40**	52 (41.9)	30 (24.2)	42 (33.9)	72 (58.1)		6 (4.8)	25 (20.2)	21 (16.9)	62 (47.3)	43 (32.8)	16 (12.2)	16 (12.2)	67 (51.1)	64 (48.9)	56 (42.7)	75 (57.3)	68 (51.9)		63 (48.1)	
***Pearson Chi-Square*** ***P-value***	0.09			0.34					0.09				0.74		0.01*		0.29			
**Middle school**	5 (50.0)	2 (20.0)	3 (30.0)	6 (60.0)	1 (10.0)		1 (10.0)	2 (20.0)	5 (50.0)	2 (20.0)	2 (20.0)	1 (10.0)	3 (30.0)	7 (70.0)	2 (20.0)	8 (80.0)	4 (40.0)	6 (60.0)		
**High school**	12 (36.4)	8 (24.2)	13 (39.4)	20 (60.0)	1 (3.0)		9 (27.3)	3 (9.1)	21 (61.8)	6 (17.6)	4 (11.8)	3 (8.8)	20 (58.8)	14 (41.2)	15 (44.1)	19 (55.9)	20 (58.8)	14 (41.2)		
**Graduates**	109 (47.0)	62 (26.7)	61 (26.3)	136 (58.4)	22 (9.4)		34 (14.6)	41 (17.6)	118 (48.8)	72 (29.8)	38 (15.7)	14 (5.8)	116 (47.9)	126 (52.)	81 (33.5)	161 (66.)	136 (56.2	106 (43.8)		
**postgraduate**	55 (48.2)	20 (17.5)	39 (34.2)	80 (70.2)	3 (2.6)		21 (18.4)	10 (8.8)	58 (47.5)	29 (23.8)	22 (18.0)	13 (10.7)	59 (48.4)	63 (51.6)	41 (33.6)	81 (66.4)	66 (54.1)	56 (45.9)		
***Pearson Chi-Square*** ***P-value***	0.38			0.06					0.62				0.41		0.48		0.73			

^*^Significant at *p*< 0.05

**Fig. 3:**
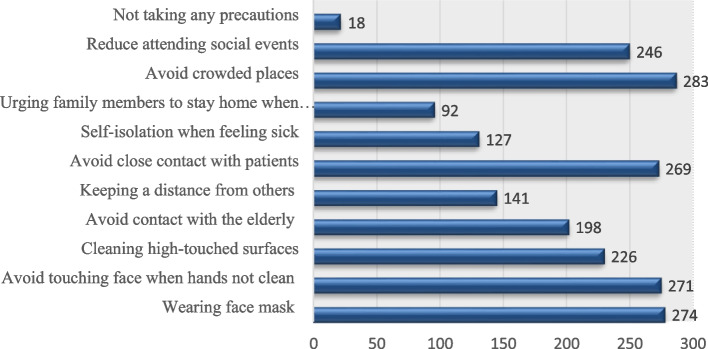
The safety practices taken by the participants

Furthermore, a limited number of the participants (18) did not take any precautionary measures to limit the virus spread and infection while, most of the participants took at least one measure. Avoiding crowded places was a common practice for the majority of the participants (*n* = 283) (Fig. 3).

The majority of the participants avoided crowded places as a common practice.

Additionally, our data showed that less than 40% of the participants did not change their attitudes towards following the precautionary measures after receiving the vaccine. A significant association was found with education levels in favour of college graduates (*p* = 0.048). Furthermore, the participants’ opinions differed regarding the effectiveness and safety of the vaccines: 37.7% were neutral; 33.1% thought that some vaccines were effective and safe; 17.4% believed all vaccines were effective and safe; and 11.8% thought all vaccines were ineffective and not safe. A significant association with gender in favour females (*p* = *0.028*) was detected. Remarkably, only a small percentage of the participants were still following updates on the pandemic (19.6%) while one-third of the respondents (33.8%) lost interest. A significant association was revealed with age in favour of > 40 years old (*p* = *0.020*).

## Discussion

The COVID-19 pandemic is not over yet, although its intensity has decreased, new cases are registered daily [16]. In Syria, according to the WHO statistics, the total number of confirmed cases reached 57,423 on 26 April 2023 [17]. Understanding how people behave during pandemics is particularly important, especially in poor countries, as this knowledge can help governments to formulate suitable strategies. In this study, we assessed the personal attitudes and practices towards the COVID-19 pandemic among a group of educated Syrian participants, following several waves of infection, and the release of different types of vaccines. The total number of individuals who volunteered in our survey was 408, generally females, between 20–29 years old, and college graduates.

The majority of the respondents (~ 86%) acknowledged being infected previously with the Coronavirus. The highest infection rates have been observed among females, with a significant association with age. Remarkably, the participants who sought medical confirmation (PCR test) were females and > 40 years old indicating greater concern, particularly amongst this group. The influence of gender on the infection rates of COVID-19 was previously researched. Higher infection risk was observed among females than males especially during peak times, due to their higher number of contacts consequent to their caregiving responsibilities at home and in the workplace, besides gender inequalities in political considerations, financial resources and access to healthcare facilities [18, 19]. This explanation may be supported by the fact that in our study, a close association was found between the infection of a family member and the recurrence of participant infection. However, the education level may have played an effective role in preventing the recurrence of the infection in most cases.

Many factors may influence vaccine acceptance [20, 21] and this may differ from one country to another and from one particular phase to another, [22, 23]. In our study, approximately half of the participants reported being vaccinated; the majority were college graduates and > 40 years old, indicating age influence on vaccine acceptance amongst this group of participants. This result is in agreement with a previous nationwide study conducted on the Syrian population where individuals between 45–65 years old expressed their desire to receive the vaccine more than others [24]. Interestingly, in our study the percentage of male participants who received vaccination was higher than that of their female counterparts. This shows that even though females were more concerned and their lives were more influenced by the pandemic, they seemed more unwilling to get vaccinated. Our results were in line with previous studies in which a significant distinction was found between males and females in their intentions to get the vaccine [24, 25].

Remarkably, a comparative study between Jordanians, Palestinians and Syrians showed that Syrians were the least interested in vaccination among other nations [26]. Furthermore, the latest statistics show that about 27% only of the Syrian population has received the vaccine [17]. This figure raises a great concern, since it is far away from reaching the accepted percentage for the herd immunity [27]. The participants differentiated between the different types of vaccines from the effectiveness and safety standpoints; only 17.4% thought that all vaccines were effective and safe, while 11.8% thought that all vaccines were ineffective. The discrepancy concerning COVID-19 vaccines in public opinion was notably observed in many studies worldwide and the rates of vaccine acceptance have changed over time, as new vaccines are developed and sides effects are reported. For example, in a comprehensive survey among physiotherapy students in the United Arab Emirates, the majority of the students believed that vaccines are safe, while nearly thirty percent did not believe in the effectiveness of the vaccine [28].

In addition, a Korean widespread analysis revealed a notable difference in public opinion for each vaccine brand [29]. Furthermore, a slight difference in opinions towards locally produced and foreign vaccines was found, among Iranian people [30].

Adherence to precautionary behaviour after vaccination differed between studies. In our study, 38.7% of the participants maintained precautionary measures after vaccination. A significant association with education in favour of college graduates was found. Our result indicates a high degree of commitment to WHO instructions among this particular group. A decline in precautionary behaviour after vaccination was also observed amongst vaccinated people in Jazan, Saudi Arabia. However, older age and females were associated with higher adherence levels [31]. By contrast, in a test on a British cohort, no decrease in precautionary behaviour among vaccinated individuals was observed [32].

Although some studies showed that education has a major influence in shaping attitudes, and practices towards COVID-19 [33, 34], in the present study, the level of education did not significantly dominate the participants’ attitudes or practices towards the COVID-19 pandemic.

Evidence from previous studies showed that the COVID-19 pandemic has impacted the lives of people worldwide. Social, physiological, economic and psychological effects were more noticed among females, as they are more vulnerable and emotional [35–38]. Our survey revealed that female participants were more worried, and the pandemic greatly affected their lives and their relationships with others. These results may be attributed to the nature of the Syrian society in which females are the caretakers of the family, besides their fears of who takes care of them and their family if they develop the infection. This explanation was supported by our other results which showed that female participants and participants > 40 years old were more committed and had positive attitudes towards self-isolation and wearing face masks when feeling sick. These results accord with a previous study conducted on a different Syrian cohort [39] and also with a study from the United States [40].

Furthermore, the impact of COVID-19 pandemic on mental health and the quality of life was observed. Stress, anxiety, and depression were common impacts in different studies [41–44]. In our study, significant differences between genders and between the age groups were detected in the anxiety level towards COVID-19. This finding accords with previous studies, in which anxiety levels were significantly affected by the female gender [45, 46]. Remarkably, only one fifth of the participants were still closely following updates on the pandemic, which may show that the pandemic is not anymore viewed as a hot topic by the general population. This data corresponds with the results from Turkey where over half of the respondents recounted not needing any additional information on COVID-19 and not being interested in a continuous update on the disease [47].

There is no doubt that taking precautionary measures and using personal protective equipment is one of the most important practices to prevent infection with the Coronavirus. This survey revealed that approximately 96% of the participants showed a high commitment to preventive measures against COVID-19 infection. Similar findings were observed in a previous study [48]. Age and female gender correlated significantly with some precautionary measures. This finding agrees with a previous study from Iran, which found that some particular practices were associated significantly with higher age, and with female gender [49].

### Strengths and limitations

This survey explored personal experiences and practices amongst educated Syrian participants, after several waves of infection and a decrease in the number of deaths and infected cases. In particular, this study is helpful in identifying the perceptions and level of COVID-19 vaccines acceptance. Future studies are required on a country-level to allow better exploration of potential factors of vaccine hesitancy and how society deals with the pandemic in different phases. However, being a cross-sectional study, some limitations may occur including the relatively small number of the participants due to low response rate and the limited number of socio-demographics beside recall biases should be considered.

## Conclusions

In the present study, the level of education did not significantly dominate the participants’ attitudes or practices towards the COVID-19 pandemic. Females especially those over the age of 40 years old were more cautious, anxious and more committed to taking the precautionary measures. However, their unwillingness to receive the vaccine raises significant concerns. Efforts should be made to emphasize the importance of immunization, the safety and efficacy of vaccines, and to promote immunization among individuals.

### Supplementary Information

**Additional file 1.**

## Data Availability

All data generated or analysed during this study are included in this article. Also, data are available from the corresponding author on reasonable request.

## Abbreviations

WHO: World Health Organization
COVID-19: Coronavirus disease of 2019
SARS-CoV-2: Severe acute respiratory syndrome coronavirus 2
PPE: Personal protective equipment
PCR: Polymerase Chain Reaction
