# Supplementary material for: Assessment of attitudes and practices towards COVID-19 pandemic: a survey on a cohort of educated Syrian population
Source: J Egypt Public Health Assoc. 2023 Sep 4;98:17. doi: 10.1186/s42506-023-00142-8 (PMC10475446; doi:10.1186/s42506-023-00142-8)
Supplement: Supplementary file 1 — Additional file 1. [file 42506_2023_142_MOESM1_ESM.docx]

**Assessment of attitudes and practices towards COVID-19 pandemic: A survey on a cohort of educated Syrian population**

**Introduction**

There is an enormous amount of information on the Coronavirus diseases (Covid-19), but people have different views of the virus which may reflect their related personal attitudes and practices towards it. We would like to know about your experience, personal opinion, attitude~~s~~ and practices towards the COVID-19 pandemic through a set of questions which will not take much of your time.

(**Answering this survey means we have your consent to participate in this study**)

**Please tick the appropriate answer**

**Q1**: Gender: Male Female

**Q2**: Age in years: Under 20 20-29 30-40 Over 40.

**Q3**: Education: Middle school High School Graduate Postgraduate

**Q4**: Have you been infected with COVID-19? Yes Maybe No

**Q5**: *If your answer is yes or maybe*, choose the period of infection from the following list. *More than one option can be selected for multiple infections:*

Beginning of the pandemic 2020 End of 2020 Beginning of 2021 Mid of 2021

End of 2021

**Q6:** Have you been infected more than once?

Yes Maybe No

**Q7**: Has the infection with COVID-19 been confirmed by lab tests (PCR)? Yes No

**Q8**: Have you suffered from any of the following symptoms during COVID-19 infection (s)? *More than one option can be selected*

High temperature Persistent cough General fatigue Diarrhoea Shortness of breath

Runny Nose Sneezing Others

**Q9**: Will you isolate yourself if you feel one or more of the previous symptoms?

Yes Maybe No As needed

**Q10**: Has the Corona pandemic affected your life and the way you deal with others?

Yes Maybe No Neutral

**Q11**: Has anyone in your home been infected with the Coronavirus? Yes Maybe No

**Q12:** *If the answer is yes,* *or maybe* how did you deal with the patient?

Cautiously Indifferently Avoided

**Q13**: How worried are you about the impact of the Coronavirus on your life?

Very worried Somewhat worried neutral Somewhat unconcerned Not worried at all

**Q14**: What is your opinion about the need to wear a face mask and its effectiveness?

Face mask must always be worn, especially in poorly ventilated and crowded places

It is not obligatory to wear a face mask, as it does not protect against the infection

I know the importance of wearing a face mask, but I'm not committed

**Q15**: If you are committed to wearing a face mask, do you cough or sneeze in it?

Yes No Sometimes

**Q16**: Do you replace or wash your face mask regularly?

Always Sometimes When it becomes dirty No

**Q17**: Before the pandemic, did you always wash your hands with soap and water for at least 20 seconds, or did you clean your hands with hand sanitiser if soap and water were not available?

Always Often Sometimes No

**Q18**: What measures/precautionary measures did you take of fear of infection and to limit the spread of the pandemic? *More than one option can be selected.*

Wearing a facemask

Avoiding touching the face when hands are not clean/sterilized

Cleaning high touch surfaces

Avoiding contact with the elderly or people with long-term health conditions

Keeping a distance from others

Avoiding close contact with people who have symptoms

Self-isolation when feeling sick

Encouraging members of family to stay at home when feeling sick

Avoiding crowded places

Reducing attending social events

Not taking any precautions

**Q19**: Have you been vaccinated /planning on getting vaccinated against the Coronavirus?

Yes Not ready yet Neutral Never

**Q20**: *If your answer was yes*, did you reduce the precautionary measures after that?

Yes Sort of No

**Q21**: In your opinion, are vaccines against the Coronavirus safe and effective?

Yes, all of them Some are safe and effective neutral All of them are unsafe and ineffective

**Q22**: Are you still following updates on the pandemic/ or have you lost interest?

Yes, eager to follow Sometimes I follow neutral I no longer care

**Thank you for your participation and your precious time**
